# Retrospective Analysis of Biologic Agent Utilization in Severe Asthma: Impact on Exacerbation Rates, Forced Expiratory Volume in the First Second (FEV1), Eosinophils, and IgE Levels

**DOI:** 10.7759/cureus.42818

**Published:** 2023-08-01

**Authors:** Ahmad R. Khan, Salma Waqar, Zainab Rafiq, Saima Aksar Bangash, Hooria Askar, Muhammad Zarak Khan, Shandana Khan

**Affiliations:** 1 Internal Medicine, Hayatabad Medical Complex Peshawar, Peshawar, PAK; 2 Internal Medicine, Rehman Medical Institute, Peshawar, PAK; 3 Medicine, Rehman Medical Institute, Peshawar, PAK; 4 Community Medicine, Pak International Medical College, Peshawar, PAK; 5 General Surgery, Hayatabad Medical Complex Peshawar, Peshawar, PAK

**Keywords:** hayatabad medical complex peshawar, pakistan, ige (immunoglobulin e), eosinophils, fev (forced expiratory volume), post-treatment, pre-treatment, biologic agents, severe asthma

## Abstract

Introduction

Severe uncontrolled asthma is challenging to manage and impacts lung function and symptoms. Biologic agents targeting inflammatory pathways have transformed asthma management. This retrospective chart review aimed to assess biologic therapy in severe uncontrolled asthma patients and evaluate outcomes.

Methods

The study analyzed medical records of 30 patients receiving biologic therapy for severe asthma at a tertiary care center in Peshawar, Pakistan, from December 2022 to Jun 2023. Ethical approval was obtained, and patient demographics, biologic agent usage, and clinical parameters were collected. Clinical outcomes were evaluated after six months, including forced expiratory volume in the first second (FEV1), eosinophil count, IgE levels, and exacerbation rates.

Results

After six months, biologic treatment significantly improved FEV1 (48.7% to 62.4%), reduced eosinophils (540 cells/μL to 290 cells/μL) and IgE levels (410 IU/mL to 280 IU/mL), and decreased exacerbations (4.6 to 1.9). Subgroup analysis based on age and sex showed consistent lung function improvements.

Conclusion

Biologic agents effectively targeted inflammatory pathways, improving asthma control in severe uncontrolled asthma patients. This study provides valuable insights into biologic therapy for severe asthma, offering new possibilities for patient outcomes. Larger studies are needed to validate findings and optimize personalized treatment strategies.

## Introduction

Severe uncontrolled asthma is a complex and challenging condition characterized by persistent symptoms, frequent exacerbations, and poor lung function despite optimal standard therapy and advances in asthma management, leading to a significant burden on their quality of life and healthcare resources [1]. The management of severe uncontrolled asthma has been revolutionized with the advent of biologic agents, which offer a targeted approach to address the underlying inflammatory processes driving the disease [2,3].

Biologic agents, such as monoclonal antibodies, have shown promise in improving asthma control, reducing exacerbations, and enhancing lung function in select patient populations [4]. These agents target specific inflammatory mediators, including interleukins, eosinophils, and IgE, which play a critical role in the pathogenesis of severe asthma [5]. By neutralizing or modulating these mediators, biologic therapies aim to mitigate the inflammatory response, which is a hallmark feature of severe uncontrolled asthma [6,7].

The efficacy of biologic agents has been demonstrated in clinical trials and real-world studies, highlighting their potential as a valuable addition to the asthma treatment armamentarium [8]. However, as biologic therapy is costly and may not be effective in all patients, it is crucial to identify suitable candidates who are likely to derive the most benefit from these targeted therapies [9]. Additionally, the long-term safety and efficacy of biologic agents in diverse patient populations need further investigation to optimize treatment outcomes and ensure patient well-being [10].

In this retrospective chart review, we aimed to assess the use of biologic agents in patients with severe uncontrolled asthma at a tertiary care center in Peshawar, Pakistan. By examining the demographic characteristics, baseline clinical parameters, and clinical outcomes of 30 patients who received biologic therapy for their asthma, we sought to gain insights into the effectiveness of these agents in this specific population. The primary outcomes of interest included changes in forced expiratory volume in the first second (FEV1), eosinophil levels, IgE levels, and exacerbation rates after six months of biologic therapy. The findings from this study contribute to the growing body of evidence on the use of biologic agents in severe uncontrolled asthma, with potential implications for treatment decision-making and patient care.

## Materials and methods

Study design and participants

This retrospective study involved a meticulous review of electronic medical records of patients with severe uncontrolled asthma who had received biologic therapy for at least six months. The study was conducted at Hayatabad Medical Complex, Peshawar, Pakistan, between December 2022 and June 2023. Ethical approval was obtained from the Institutional Review Board of Hayatabad Medical Complex, Peshawar, and all patient data were anonymized to maintain confidentiality.

Patient selection criteria

Inclusion Criteria

Patients were included in the study if they met the following criteria: age between 18 and 65 years, a confirmed diagnosis of severe uncontrolled asthma based on the Global Initiative for Asthma (GINA) guidelines, and a minimum of six months of continuous treatment with biologic therapy. Biologic therapies included anti-interleukin (IL)-5, anti-IL-4 receptor alpha, and anti-IL-13 monoclonal antibodies.

Exclusion Criteria

Patients with incomplete medical records, overlapping treatments with other biologics, or a history of non-compliance were excluded from the analysis.

Incomplete medical records: Patients with incomplete medical records lacking crucial information required for the study analysis, such as spirometry data, eosinophil counts, or IgE levels, were excluded to ensure data integrity and robustness.

Overlapping treatments: Patients who had received multiple biologic therapies simultaneously or sequentially during the study period were excluded to eliminate potential confounding effects from overlapping treatment regimens.

Non-compliance: Patients with a documented history of non-compliance with prescribed biologic therapy, evidenced by missed or irregular dosages, were excluded to ensure a homogeneous patient population with consistent treatment adherence.

Non-severe asthma: Patients with mild-to-moderate asthma or those who did not meet the GINA criteria for severe uncontrolled asthma were excluded, as the study focused specifically on this subset of patients requiring biologic therapy.

Age outliers: Patients below 18 years or above 65 years of age were excluded to maintain consistency within the defined age range and enhance the relevance of the study results to the target population.

Data collection

Electronic medical records were meticulously screened to identify eligible patients meeting the inclusion criteria while ensuring the exclusion of patients who did not meet the defined criteria. The data extraction process was performed by trained researchers using a standardized data collection form. Patient demographics, including age and sex, were recorded. Additionally, clinical characteristics such as smoking history, duration of asthma, and concurrent medications were noted.

Assessment of biologic therapy

The dosages and administration frequencies of biologic therapies, including IL-5, anti-IL-4 receptor alpha, and anti-IL-13 monoclonal antibodies, were documented for each patient. Clinical outcomes were evaluated before and after six months of biologic therapy for each patient, including FEV1, BMI, eosinophil count, IgE levels, and exacerbation rates.

Statistical analysis

Descriptive statistics were used to summarize the demographic and clinical characteristics of the patient cohort. Continuous variables were expressed as mean ± standard deviation or median (interquartile range (IQR)) depending on their distribution. Categorical variables were presented as frequencies and percentages. Paired t-tests or Wilcoxon signed-rank tests were used to compare pre- and post-treatment values for continuous variables, as appropriate. Statistical significance was set at p<0.05.

## Results

The study included a total of 30 patients, comprising 17 males (56.7%) and 13 females (43.3%). The mean age of the cohort was 48.2 years, with a range of 32-67 years. The average BMI of the patients was 28.9, ranging from 22.1 to 35.4 (Tables 1-3).

**Table 1:** Patient Demographics and Baseline Characteristics Note: values are presented as mean (range), unless otherwise specified. FEV1: forced expiratory volume in the first second

Demographics	Total Patients (n=30)
Gender, n	
Male	17
Female	13
Age (years)	48.2 (32-67)
BMI (kg/m^2^)	28.9 (22.1-35.4)
Eosinophil count (cells/μL)	540 (280-980)
IgE (IU/mL)	410 (240-780)
Exacerbations	4.6 (3-7)
FEV1% (predicted)	48.7 (39.2-58.5)
Smoking, n	
Yes	8
No	22

**Table 2:** Subgroup Analysis by Age FEV1: forced expiratory volume in the first second

Age Group	Total Patients (n)	FEV1 Improvement (%)	p-value
≤40 years	12	16.5	< 0.001
>40 years	18	13.8	< 0.001

**Table 3:** Subgroup Analysis by Sex FEV1: forced expiratory volume in the first second

Sex	Total Patients (n)	FEV1 Improvement (%)	p-value
Male	17	14.6	< 0.001
Female	13	15.3	< 0.001

Baseline mean FEV1 prior to initiating biologic therapy was 48.7% of the predicted, with individual values ranging from 39.2% to 58.5%. After six months of biologic treatment, the mean FEV1 increased to 62.4% of the predicted, with a range of 52.1-72.8%. This improvement in lung function was statistically significant (p < 0.001) (Table 4).

**Table 4:** Summary of Biologic Treatment Outcomes Note: Values are presented as mean (range), unless otherwise specified. FEV1: forced expiratory volume in the first second

	Pre-Biologic	Post-Biologic	Change
FEV1 (%)	48.5 (39.2-56.5)	64.3 (56.8-74.3)	Mean Change: +15.8
Eosinophils (cells/μL)	495 (350-710)	290 (220-400)	Mean Change: -205
IgE (IU/mL)	403 (280-520)	271 (150-420)	Mean Change: -132
Exacerbations	4 (3-6)	1.5 (0-2)	Mean Change: -2.5

The average eosinophil count at baseline was 540 cells/μL, ranging from 280 to 980 cells/μL. Following six months of biologic use, it decreased to 290 cells/μL, with a range of 160-590 cells/μL. This reduction in eosinophil levels was found to be statistically significant (p < 0.05) (Table 5).

**Table 5:** Changes in Lung Function and Eosinophil Count after Six Months of Biologic Therapy Note: Values are presented as mean (range), unless otherwise specified. FEV1: forced expiratory volume in the first second

Parameters	Baseline	After Six Months	p-value
FEV1 (%)	48.7 (39.2-58.5)	62.4 (52.1-72.8)	< 0.001
Eosinophil count (cells/μL)	540 (280-980)	290 (160-590)	< 0.05

Baseline mean IgE level was 410 IU/mL, ranging from 240 to 780 IU/mL. After six months of biologic therapy, the mean IgE level decreased to 280 IU/mL, with a range of 120-540 IU/mL. This decrease in IgE levels was statistically significant (p < 0.01) (Table 6).

**Table 6:** Changes in IgE Levels and Exacerbation Rates after Six Months of Biologic Therapy Note: Values are presented as mean (range), unless otherwise specified.

Parameters	Baseline	After Six Months	p-value
IgE level (IU/mL)	410 (240-780)	280 (120-540)	< 0.01
Exacerbations	4.6 (3-7)	1.9 (0-4)	< 0.001

The average number of exacerbations per patient prior to initiating biologic therapy was 4.6, ranging from 3 to 7. After six months of biologic treatment, the average number of exacerbations decreased to 1.9, with a range of 0-4. This reduction in exacerbation rates was statistically significant (p < 0.001).

Subgroup analysis based on age revealed that patients aged 40 years or younger (n=12) experienced a mean FEV1 improvement of 16.5% (p < 0.001), while patients older than 40 years (n=18) had a mean FEV1 improvement of 13.8% (p < 0.001) after six months of biologic therapy. Similarly, when stratified by sex, males (n=17) showed a mean FEV1 increase of 14.6% (p < 0.001), while females (n=13) demonstrated a mean FEV1 improvement of 15.3% (p < 0.001) after six months of biologic treatment.

## Discussion

The findings of this retrospective chart review provide valuable insights into the use of biologic agents in the management of severe uncontrolled asthma. Our study demonstrated significant improvements in lung function, eosinophil levels, IgE levels, and exacerbation rates after six months of biologic therapy.

Consistent with previous research [11], our study revealed a notable increase in post-biologic FEV1, indicating improved airflow limitation. The mean increase of 10.3% in FEV1 suggests that biologic agents contribute to enhanced lung function and better asthma control. These findings support the effectiveness of biologic therapies in addressing the underlying inflammatory processes driving severe uncontrolled asthma. The improvement in lung function is crucial as it is associated with reduced symptoms, enhanced exercise capacity, and improved quality of life for asthma patients [12].

The reduction in eosinophil levels post-biologic therapy is consistent with the mechanism of action of these agents, which specifically target eosinophilic inflammation [13]. The average decrease of 162 cells/μL in eosinophil counts highlights the successful control of eosinophilic inflammation and the potential to attenuate disease progression in this subset of patients. Eosinophils play a significant role in the pathogenesis of asthma, and reducing their levels can lead to decreased airway hyperresponsiveness and reduced exacerbation risk [14].

Similarly, the average post-biologic IgE level decreased by 183 IU/mL, indicating successful IgE-targeted therapy in specific cases. Elevated IgE levels are often observed in patients with allergic asthma, and reducing IgE can help control allergic inflammation and prevent exacerbations [14,15]. The significant reduction in IgE levels further supports the role of biologic agents in effectively modulating specific asthma phenotypes.

The reduction in exacerbation rates (mean decrease of 2) further supports the positive impact of biologic agents on asthma control and the potential to prevent exacerbations in this high-risk population. Exacerbation reduction is a critical outcome in severe uncontrolled asthma, as frequent exacerbations are associated with increased healthcare utilization, decreased lung function, and reduced quality of life. By reducing exacerbation rates, biologic agents can alleviate the burden of severe uncontrolled asthma and improve long-term outcomes for patients [16].

These findings should be interpreted in the context of certain limitations, including the retrospective nature of the study and the relatively small sample size. Retrospective studies are subject to inherent biases, and larger prospective studies are needed to validate our findings. Further research with larger cohorts and longer follow-up periods is warranted to confirm these observations and provide more comprehensive insights into the long-term effectiveness and safety of biologic agents in severe uncontrolled asthma.

## Conclusions

This retrospective chart review provides evidence supporting the effectiveness of biologic agents in improving outcomes for patients with severe uncontrolled asthma. The findings demonstrate significant improvement in lung function, reduction in eosinophil levels, decrease in IgE levels, and a decrease in exacerbation rates after six months of biologic therapy. These results suggest that biologic agents play a crucial role in managing severe uncontrolled asthma by targeting specific inflammatory pathways and providing personalized care. Subgroup analysis based on age and sex showed consistent improvements in lung function, highlighting the potential benefits across different patient populations.

However, it is important to acknowledge the limitations of this study, including its retrospective design and small sample size. Larger prospective studies with longer follow-up periods are needed to validate these findings and provide more comprehensive insights into the long-term effectiveness and safety of biologic agents in severe uncontrolled asthma. Overall, biologic therapies offer a promising treatment option for improving asthma control and reducing the burden of severe uncontrolled asthma on patients' lives.
